# Perlecan expression influences the keratin 15‐positive cell population fate in the epidermis of aging skin

**DOI:** 10.18632/aging.100928

**Published:** 2016-03-17

**Authors:** Morgan Dos Santos, Anna Michopoulou, Valérie André‐Frei, Sophie Boulesteix, Christine Guicher, Guila Dayan, John Whitelock, Odile Damour, Patricia Rousselle

**Affiliations:** ^1^ Laboratoire de Biologie Tissulaire et Ingénierie Thérapeutique, UMR 5305, CNRS, University Lyon 1, Lyon, France; ^2^ BASF BCS France SAS, 69366, Lyon, France; ^3^ Graduate School of Biomedical Engineering, University of New South Wales Sydney, Australia; ^4^ Cell and Tissue Bank, Hôpital Edouard Herriot, Lyon, France

**Keywords:** basement membrane, perlecan, keratinocyte, skin aging, keratin 15, stem cell

## Abstract

The epidermis is continuously renewed by stem cell proliferation and differentiation. Basal keratinocytes append the dermal‐epidermal junction, a cell surface‐associated, extracellular matrix that provides structural support and influences their behaviour. It consists of laminins, type IV collagen, nidogens, and perlecan, which are necessary for tissue organization and structural integrity. Perlecan is a heparan sulfate proteoglycan known to be involved in keratinocyte survival and differentiation. Aging affects the dermal epidermal junction resulting in decreased contact with keratinocytes, thus impacting epidermal renewal and homeostasis. We found that perlecan expression decreased during chronological skin aging. Our in vitro studies revealed reduced perlecan transcript levels in aged keratinocytes. The production of in vitro skin models revealed that aged keratinocytes formed a thin and poorly organized epidermis. Supplementing these models with purified perlecan reversed the phenomenon allowing restoration of a well‐differentiated multi‐layered epithelium. Perlecan down‐regulation in cultured keratinocytes caused depletion of the cell population that expressed keratin 15. This phenomenon depended on the perlecan heparan sulphate moieties, which suggested the involvement of a growth factor. Finally, we found defects in keratin 15 expression in the epidermis of aging skin. This study highlighted a new role for perlecan in maintaining the self‐renewal capacity of basal keratinocytes.

## INTRODUCTION

Skin constitutes the protective barrier of the body and mediates its interaction with the environment. A highly specialized extracellular matrix (ECM) named dermal epidermal junction (DEJ) separates the epidermal and dermal compartments. The epidermis, primarily made of keratinocytes, is continuously renewed by the proliferation of stem cells and the differentiation of their progeny, which undergo terminal differentiation as they leave the basal layer and move upward toward the surface, where they die and slough off [1]. Basal keratinocytes append the DEJ, a cell surface-associated extracellular matrix that forms as a concerted action of both epidermal and dermal cells [2]. The DEJ provides both a structural support to keratinocytes and a specific niche that mediates signals influencing their behaviour. Similar to all basement membranes (BMs), the DEJ primarily consists of laminins, type IV collagen, nidogens, and the heparan sulfate proteoglycan (HSPG) perlecan, all of which are necessary for tissue organization and structural integrity. Keratinocytes committed to the differentiation program downregulate integrins to become less adhesive, move to the suprabasal compartment and continue their upward movement until they are terminally differentiated and shed off. This produces several layers of keratinocytes, at different stages of differentiation that can be identified by the expression of keratins. Basal keratinocytes express keratins (K) 5, K14 and K15, whereas differentiating keratinocytes express keratins K1 and K10 [3].

The appearance and mechanical functions of skin undergo profound changes over time with both increasing chronological age and cumulative exposure to external factors, such as ultraviolet radiation [4]. In addition to an overall thinning of the epidermis and dermis, a major change is the flattening of the DEJ resulting from retraction of both epidermal papillae and microprojections of basal cells into the dermis [5,6]. In genetically programmed aging, the progressive decrease in epidermal cell renewal results from decreased proliferation [7,8] and the appearance of senescent keratinocytes [9].

**Figure 1:**
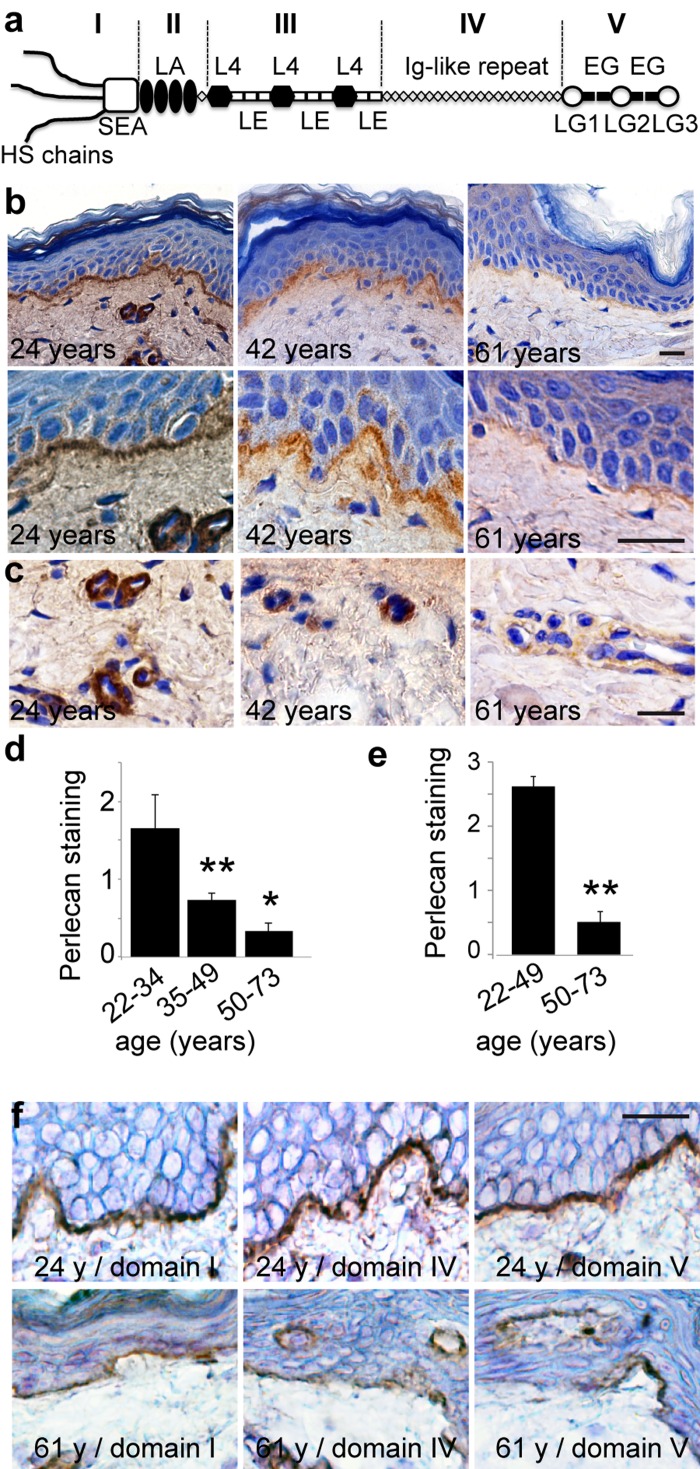
Age‐related changes in perlecan expression in skin basement membranes (**a**) Schematic representation of perlecan. (**b, c**) Perlecan expression in skin from women donors aged 24, 42, and 61 years (domain III stain). Sections of paraffin‐embedded skin biopsies were stained for perlecan and counterstained with Harris haematoxylin. (**d, e**) Quantification of perlecan staining in (d) epidermal and (e) vascular BMs in 38 human skin samples from donors of ages ranging from 22 to 73 years (22‐33, n=8; 35‐49, n=12; 50‐73, n=18). Mean ± SD, ^**^p<0.01 vs. young group, based on Student's t‐test. (**f**) Age‐related reductions in perlecan subdomains. Sections of frozen skin from donors aged 24 and 61 years were immunolabelled with antibodies specific for perlecan domain I, domain IV, or domain V, and counterstained with Harris haematoxylin. (**b, c, f**) *Scale bar* = 20 μm

In addition to their function in the promotion of strong epidermal/dermal attachment, DEJ components play crucial roles in biological events, including the adhesion, migration, proliferation and differentiation of keratinocytes [2]. In particular, perlecan was shown to play a role in keratinocyte survival and terminal differentiation [10] and in DEJ cohesion [11]. Perlecan is a modular HSPG with broad tissue distribution and multiple functions [12]. The 470-kDa perlecan core protein is made of five distinct modules, but together with several O-linked oligosaccharides and heparan sulfate chains, the protein can reach a molecular mass of over 800 kDa (Figure 1a). The various protein modules of perlecan and its heparan sulfate chains take part in numerous interactions with partners, including growth factors, BM constituents such as laminin-111 or -511 and collagen IV, as well as Δ1 integrins [13,14]. Though both epidermal keratinocytes and dermal fibroblasts express perlecan at the transcriptional level, DEJ associated perlecan seems to be produced by keratinocytes [10,15].

In the present study, we examined the expression profile of perlecan during chronological skin aging. We found decreased expression in the epidermal and microvessel BMs. Our *in vitro* studies with keratinocytes from aged donors confirmed these findings and also indicated reduced levels of perlecan transcription. The use of *in vitro* skin models comprising epidermal keratinocytes from young and elderly donors confirmed earlier studies showing that perlecan influences epidermal thickness [10]. Finally, we found that perlecan down-regulation in keratinocytes resulted in the depletion of the cell population that expressed keratin 15 and Δ1-integrin. This depletion was reversed when we supplemented perlecan-deficient keratinocytes with purified perlecan.

## RESULTS

### Perlecan expression in epidermal and capillary BMs in skin aging

We performed an immunohistochemical analysis of perlecan in a cohort of 38 human skin samples from donors ranging in age from 22 to 73 years. We detected perlecan expression with a monoclonal antibody (mAb) against perlecan domain III (Figure 1a). An analysis of the biopsies from donors that were 22 to 35 years old revealed continuous staining at the DEJ and around dermal capillaries (Figure 1b and c), consistent with previous studies [16,17]. Perlecan staining began to decrease in biopsies from donors aged 39 to 50 years. Perlecan staining again decreased in both intensity and area in biopsies from donors aged 54 to 70 years (Figure 1b and d). This was also observed in the capillary BMs (Figure 1c and e). An analysis of perlecan domains I, IV, and V revealed that all domains were expressed along the DEJ and around dermal capillaries, similar to the domain III expression pattern (Figure 1f). In aged skin, both the DEJ and dermal capillary BM showed reduced staining of each domain; this result suggested that the entire perlecan molecule was subject to expression changes over time.

**Figure 2:**
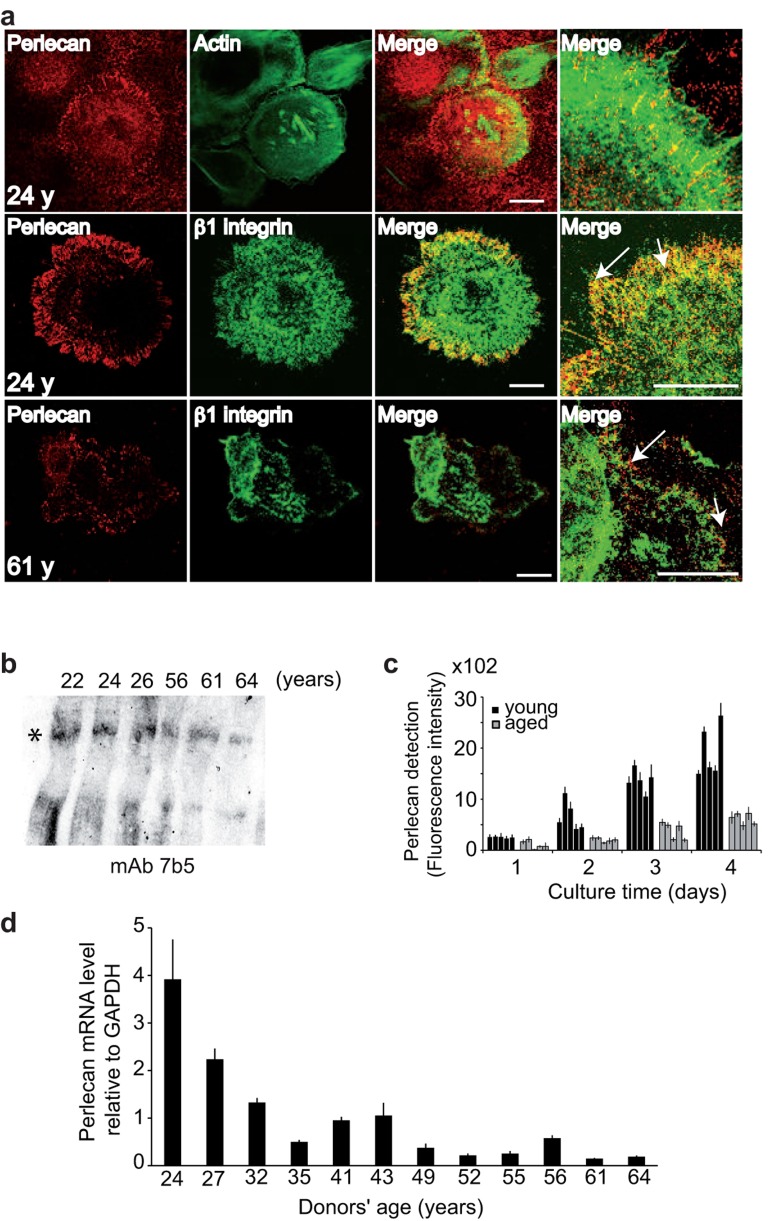
Keratinocyte aging results in decreased perlecan in the ECM (**a**) NHKs from young or aged donors were cultured, fixed, and stained with either polyclonal antibodies (pAb) against perlecan (red), phalloidin‐FITC (green) or monoclonal antibodies (mAb) against Δ1‐integrin (green), as indicated. The merged images show the juxtaposition of perlecan and β1‐integrin staining (arrows). Optical slices (0.8 μm) were acquired at the cell‐matrix interface. *Scale bar* = 50 μm. (**b**) Perlecan immunoblotting in the ECM. Ages are indicated, and the asterisk shows perlecan. (**c**) Perlecan quantification. NHKs (5 × 10^3^) from donors aged 22, 24, 26, 27, and 32 years or 52, 55, 56, 61, and 64 years were seeded in 96‐well plates, and perlecan immunodetection was performed over 4 days. Six assay points were performed per experiment. (**d**) Results from QPCR of NHK cDNA show perlecan gene transcription in 12 NHK strains from donors aged 24 to 64 years, as indicated. Each assay point was performed in triplicate (**c, d**). Data are presented as mean ± SD of 3 independent experiments conducted with keratinocytes from each individual donor.

To characterize the perlecan expression pattern in cultured keratinocytes, we first examined its localization in the ECM of young keratinocyte cultures (Figure 2a). When the anti-perlecan mAb was applied to confluent cells, the protein appeared to be regularly distributed over the entire support, which suggested that perlecan was present in the underlying ECM. At the individual cell level, we observed that the substrate immediately adjacent to the cells was fluorescently stained in regularly aligned patches, resembling adhesion contacts. Higher magnification revealed that the ends of actin cables often colocalized with perlecan, which suggested that perlecan may be involved in keratinocyte adhesion. Moreover, immunostaining of β1-integrin subunits revealed that these molecules often co-localized with perlecan. Although a direct interaction remains to be demonstrated, this finding indicated that an integrin that included a β1 subunit might be involved in keratinocyte adhesion to perlecan. In comparison, an analysis of aged keratinocytes revealed similar, though much weaker, perlecan staining. Moreover, at the point of perlecan co-localization with actin, the β1-integrin subunit appeared as a faint punctuation. These results showed that perlecan expressed in keratinocyte ECM was localized in close vicinity to the cell membrane. This location was reminiscent of the previously described “cellular perlecan”. Our results also suggested that this interaction weakened with aging.

A western blot analysis of perlecan in the ECM of confluent cells from young and aged donors revealed a band of 800 kDa in all samples, which corresponded to the full-length perlecan with intact heparan sulfate (HS) chains (Figure 2b). Because the intensity appeared to slightly decrease with age, we quantified perlecan in 5 young and 5 aged keratinocyte strains (Figure 2c) as a function of cell density to circumvent the bias caused by differences in cell proliferation rates. To that end, we quantified perlecan expression in the ECM of normal human keratinocytes (NHKs) over 4 days with concomitant monitoring of cell proliferation (Additional file 1: Figure S1). The fluorescence intensity of perlecan increased with time in the group of NHKs from young donors, during both cell proliferative and confluent phases. This result showed that perlecan was continuously produced and accumulated in the ECM. In contrast, low levels of perlecan were detected in the NHKs from aged donors. Additionally, the fluorescence intensity detected in the ECM of the NHKs from aged donors on Day 4 was at least 60% lower than that detected in the NHKs from young donors. This finding suggested that less perlecan may be produced and/or retained in the ECM as cells age. To address whether an age-associated decrease in perlecan was linked to genomic breakdown, we generated cDNA from a large panel of primary NHKs obtained from skin donors aged 24 to 64 years old for real-time polymerase chain reaction (QPCR) analyses. As shown in Figure 2(d), the perlecan mRNA levels were reduced in the older age groups. A 75% decrease in perlecan mRNA expression was observed from 24 to 43 years of age, followed by an additional 12% decrease from 49 to 64 years of age. These reductions were consistent with our findings that the perlecan protein levels decreased in aging skin (Figure 1b). These results suggested that perlecan expression in keratinocytes was down-regulated at the transcriptional level without excluding a potential additional post-transcriptional regulation.

### Perlecan regulates K15 expression in cultured keratinocytes

**Figure 3:**
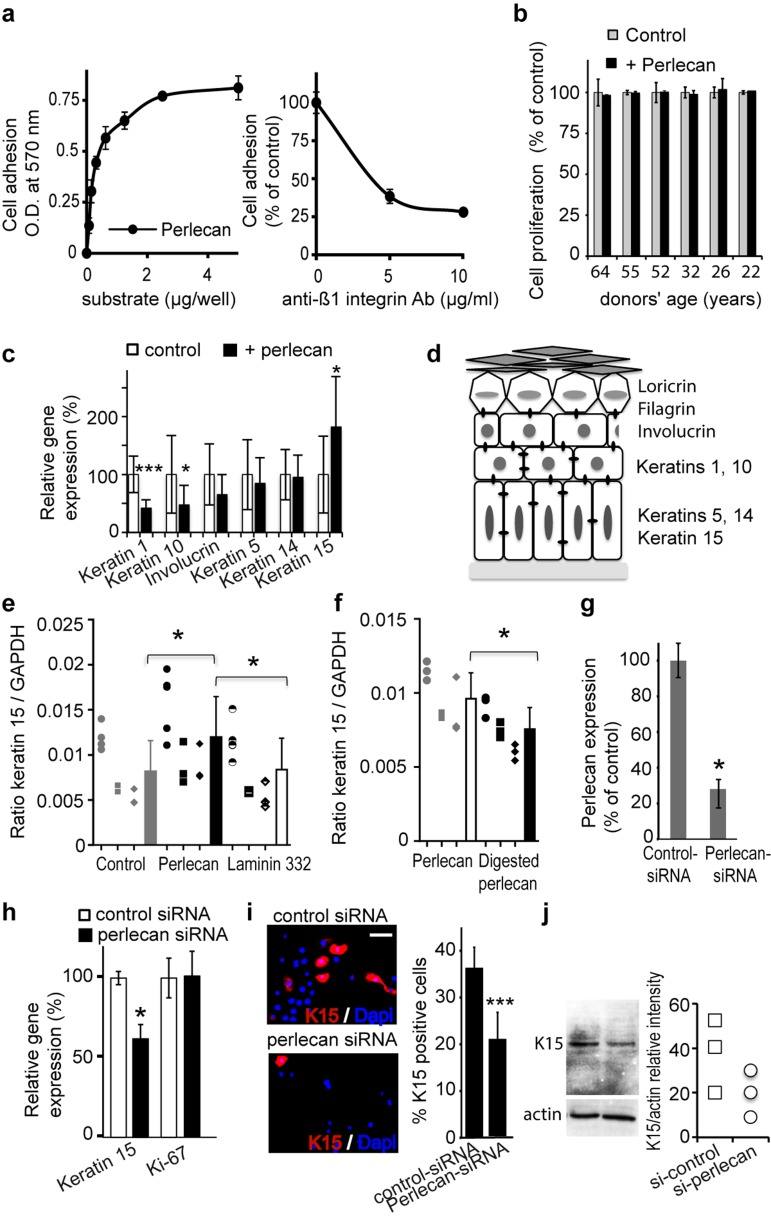
Perlecan induces keratinocyte adhesion and regulates K15 gene (KRT15) expression (**a**) Keratinocytes adhere to perlecan in a dose‐dependent manner (left). Adhesion to perlecan is inhibited by antibodies against the Δ1 integrin subunit (right). (**b**) Proliferation of aged and young keratinocyte strains over 24 h. The proliferation of cells plated on perlecan is expressed as a percentage of the proliferation observed in control conditions. (**c**) Real‐time PCR analysis of keratins (K1, K5, K10, K14, and K15) and involucrin gene expression normalized to GAPDH in keratinocytes from the 61‐year‐old donor plated on culture dishes either covered beforehand with perlecan or untreated. (d) Scheme of the epidermis showing the locations of the differentiation markers. (**e**) Real‐time PCR analysis of KRT15 in keratinocytes from 3 distinct aged donors (age 64, 61, 60), cultured on perlecan, laminin 332 or plastic. (**f**) Real‐time PCR analysis of KRT15 in keratinocytes from 3 distinct aged donors (age 64, 61, 60) cultured either on native perlecan or digested perlecan, which lacked heparin sulfate (HS) moieties. (**g**) ELISA results show expression levels of perlecan in the ECM of keratinocytes transfected with perlecan‐specific siRNA, compared to cells transfected with control‐siRNA. (**h**) Real‐time PCR analysis of KRT15 and Ki‐67 gene (MKI67) in a 52 year‐old keratinocyte strain transfected with perlecan‐specific siRNA. (**i**) K15 staining in perlecan‐siRNA‐transfected keratinocytes; quantification is expressed as a percentage of DAPI positive cells. Each slide was performed in triplicate; 400 cells were counted within 4 areas of each slide. (**j**) Western blot of K15 expression in cultured perlecan knock‐down keratinocytes. Quantification of K15 expression is relative to actin expression. Experiment was done in triplicate. (c, g, h) Data are presented as the mean ± SD of 9 independent experiments with keratinocytes from a 61 (c) or 52 (g, h) years old donor. (e, f) All performed independent experiments per donor are shown (age 64 ○, 61 □, 60 ◇) and were combined for statistical analysis. ^*^p<0.025,^**^p<0.005,^***^p<0.0002 vs. control, Student's t‐test.

Next, we evaluated perlecan cell adhesion activity. We found that purified perlecan induced adhesion of aged keratinocytes in a dose-dependent manner and that adding antibodies that specifically blocked Δ1 integrin function inhibited the adhesion of keratinocytes to perlecan. This finding suggested that a Δ1 integrin was involved in the adhesion mechanism (Figure 3a).

Therefore, we used purified perlecan as an adhesion substrate to analyse its potential impact on keratinocyte proliferation and differentiation. First, we observed that culturing keratinocytes for 24 h on immobilized perlecan did not impact their proliferation compared to control cells plated directly on the culture dishes (Figure 3b). Moreover, we found that plating aged keratinocytes on perlecan for 24 h resulted in significantly reduced mRNA expression of the early and intermediate differentiation genes K1, K10, and involucrin; however, mRNA expression of the basal keratinocyte keratins, K5 and K14, remained unchanged (Figures 3c and 3d). Most interestingly, a significant increase in the K15 mRNA level was detected in aged cells plated on perlecan, compared to controls (Figure 3c). The experiment was repeated with strains of three distinct donors, and similar results were produced (Additional file 2: Figure S2). In contrast, plating cells on the BM protein, laminin 332, did not affect the K15 mRNA expression level (Figure 3e).

In the epidermis, K15-expressing cells belong to the stem cell population known to preferentially express the β1-integrin subunit [Figure 3d; 18, 19]; therefore, we also evaluated β1-integrin mRNA expression under the same conditions (Additional file 3: Figure S3). We found that culturing four aged keratinocyte strains on perlecan for 24 h resulted in a significant increase in β1- integrin mRNA levels; this result suggested that perlecan promoted enrichment of the cell population that expressed keratin 15 and β1-integrin. Digestion of perlecan to remove the HS moieties prior to the experiment abolished the rise in K15 mRNA expression (Figure 3f). This result suggested that the HS played a role in this enrichment mechanism. We further assessed the direct role of perlecan in K15 expression by blocking perlecan expression with a combination of three siRNA oligonucleotides specific for human perlecan. Transfection of NHKs with this siRNA mixture inhibited perlecan expression by approximately 80%, based on ELISA detection of perlecan protein expression (Figure 3g). Consistent with our previous results, we also found a significant reduction in K15 mRNA expression levels in cells transfected with perlecan-specific siRNA (Figure 3h). As expected, and as revealed by the unchanged expression of the Ki-67 proliferation marker, the perlecan deficiency did not affect keratinocyte proliferation. These results were confirmed by the reduced number of K15-positive cells found in the population of perlecan-deficient keratinocytes. After knocking down perlecan expression, the ratio of K15-positive keratinocytes:total NHK cells dropped from 36.6% (control cultures) to 18.8% (perlecan-deficient cultures; Figure 3i); this result was reinforced by western blot results, which showed a similar pattern in K15 protein expression (Figure 3j). Taken together, our results revealed a link between perlecan and K15 expression in NHKs. Moreover, the finding that this link depended on perlecan HS moieties suggested the involvement of a heparin-binding partner.

**Figure 4:**
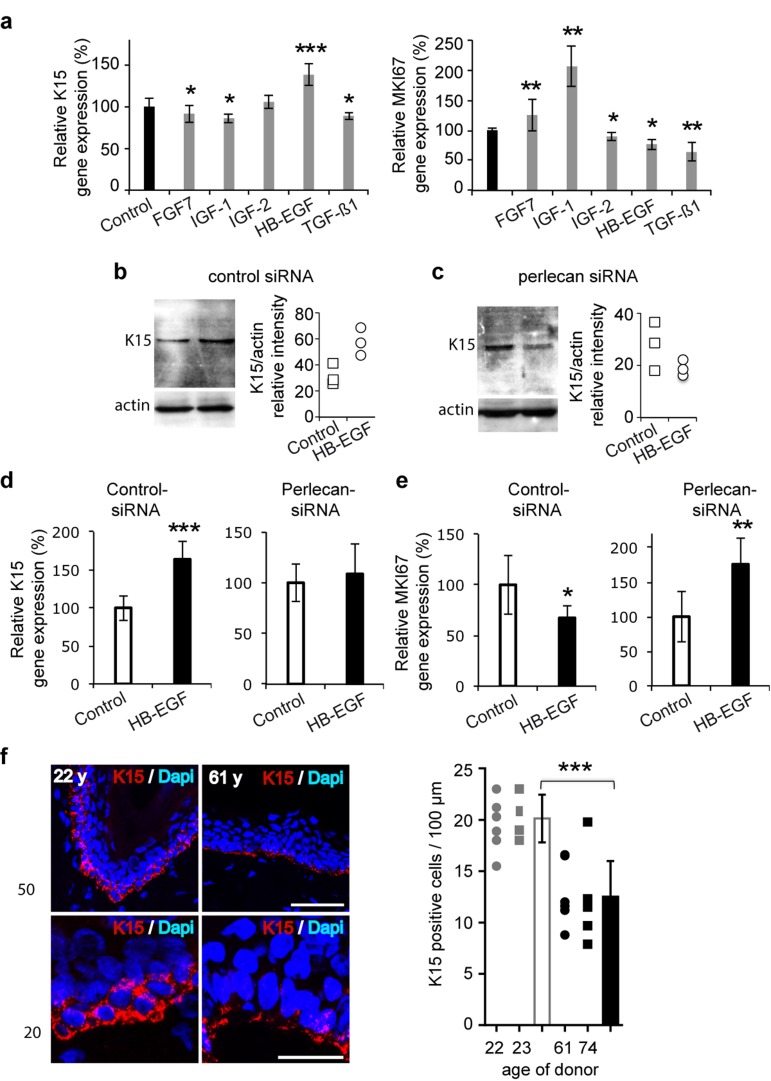
Perlecan functions in K15 expression regulation in aging skin (**a**) Real time PCR analysis of KRT15 and MKI67 in the 52‐year old keratinocyte strain after treatment with growth factors (FGF7, 15 ng/ml; IGF‐1, 50 ng/ml; IGF‐2, 80 ng/ml; HB‐EGF, 100 ng/ml; TGF‐Δ1, 1 ng/ml). (**b**,**c**) Western blot of K15 expression in control (**b**) and perlecan knock‐down (**c**) keratinocytes after HB‐EGF treatment (100 ng/ml). Quantifications of K15 expression in control‐siRNA and perlecan‐siRNA transfected cells are relative to actin expression. Experiment was done in triplicate. (d) Real time PCR analysis of KRT15 and (**e**) MKI67 in control and perlecan knock‐down keratinocytes after HB‐EGF treatment (100 ng/ml). (**f**) K15 (red) immunostaining and DAPI (blue) staining in 2 young and 2 aged skin biopsy sections. Red and blue cells were counted along the BM of 6 independent sections. *Scale bars* = 50 μm (top) and 20 μm (bottom). (**a, d, e**). Data are representative of at least 12 independent experiments with keratinocytes from the 52 years old donor. Mean ± SD; ^*^p<0.025,^**^p<0.005, ^***^p<0.0005 vs. control, Student's t‐test.

Perlecan is known to bind to many growth factors, including FGF7, which plays an important role in keratinocyte survival [10]. Therefore, we analysed FGF7, and other selected growth factors potentially involved in epidermal differentiation, to determine whether they impacted K15 expression. Simultaneously, we also verified their expected impact on keratinocyte proliferation. As previously reported, we found a slight, but significant, reduction in K15 mRNA expression levels in cells treated with either FGF7 or transforming growth factor beta 1 (TGF-Δ1) (Figure 4a) [20]. Among the other tested growth factors, we found a reduction or no change in K15 gene expression after treating with insulin growth factor (IGF)-1 or -2, respectively, and a very significant increase after treating with heparinbinding epidermal growth factor (HB-EGF). To determine whether perlecan was involved in HB-EGFinduced K15 expression, we treated perlecan knockdown cells with HB-EGF. Western blotting (Figure 4c) experiments confirmed that K15 expression was increased in control-siRNA transfected cells, and K15 expression was decreased in perlecan-siRNA transfected cells. The QPCR analysis, which confirmed these results, also drew our attention to the changes induced by HB-EGF treatment. We found that HB-EGF promoted diametrically opposite keratinocyte proliferation signals, depending on perlecan expression (Figure 4d, Additional file 4 : Figure S4). These results revealed that perlecan played a role in HB-EGFmediated regulation of proliferation and K15 expression in basal keratinocytes.

### K15 expression decreases in aging skin

We next investigated whether skin aging might affect K15 expression. We analyzed the K15-positive cell population in epidermis biopsies from four donors, aged 22, 24, 61, and 74 years. In younger specimens, as previously described, K15 was detected throughout the basal layer, and strong staining was observed in reteridges, where it was detected throughout the entire cell cytoplasm (Figure 4f) [19,21]. In contrast, in older specimens, where the epidermal rete ridges were missing, the K15 staining appeared punctuated; staining was located mainly on the basal side of cells (Figure 4f). In addition, we evaluated the density of K15-expressing keratinocytes. We found K15-positive cells at densities of 20 and 21 cells/100 μm in the epidermal rete-ridges of young specimens; the density dropped to 12 and 11/100 μm in the two tested aged specimens. These results suggested that K15 expression was decreased and disorganized in the basal keratinocytes of aged skin.

### Exogenous perlecan restores the ability of aged keratinocytes to form a full thickness epidermis

**Figure 5:**
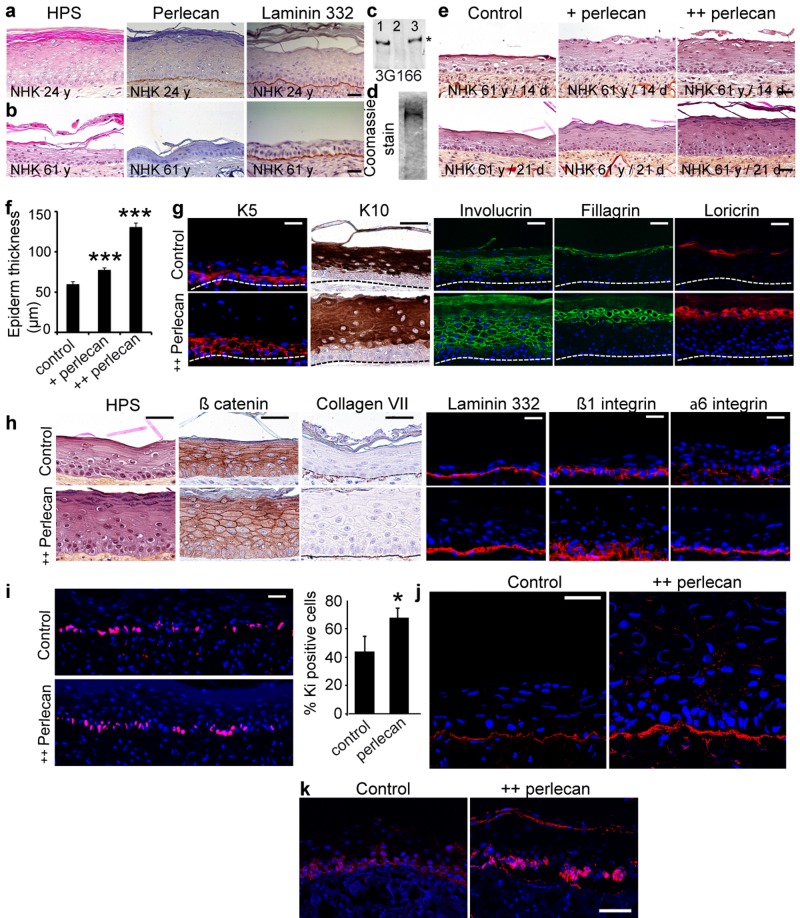
Exogenous soluble perlecan improves epidermal thickness (**a, b**) HPS staining and immunohistochemical analysis of paraffin‐embedded SEs epidermalized with (**a**) young or (**b**) aged keratinocytes. (**c**) Western blot of HUAEC culture medium before (lane 1) and after (lane 2) immunopurification. The eluted material (lane 3) contained the perlecan core protein. (**d**) Coomassie blue staining of purified perlecan. (e) SEs generated with aged keratinocytes without (control) or with perlecan (+ perlecan and ++ perlecan). SEs were collected 14 and 21 days post‐epidermalization and processed for histology. (**f**) Epidermal thickness. *** p<0.001 for + perlecan and ++ perlecan vs. control, Student's t‐test. (**g, h**) Paraffinembedded and frozen SEs at 21 days post‐epidermalization were immunolabelled to identify (g) differentiation markers and (**h**) adhesion/ECM components. Nuclei were stained with DAPI. (**i**) Ki‐67 staining in SEs. Quantification is expressed as the percentage of DAPI staining in the basal layer. Mean ± SD; *p<0.025. (j, k) Frozen SEs at 21‐days post‐epidermalization were immunostained to identify perlecan (**j**) and K15 (**k**). *Scale bars* = 50 μm (**j**), = 60 μm (**k**).

Finally, to analyze the impact of keratinocyte aging on perlecan expression in three-dimensional conditions, *in vitro* skin equivalent (SE) models were produced. These SEs were composed of a previously described dermal equivalent (DE) made of foreskin dermal fibroblasts seeded in a porous dermal substrate (collagen, glycosaminoglycans, and chitosan), on top of which NHK were seeded [22]. As shown in Figure 5a, when 24 year old donor's NHK were seeded, the DE supported epidermal proliferation giving rise to a full thickness and well organized, terminally differentiated epidermis at day 21 that displayed perlecan expression evenly along the DEJ. As previously documented [22], normal differentiation stages were indicated by expression of the early differentiation marker K10 in the supra-basal layers, the intermediate differentiation marker involucrin in the spinous layer, and the late differentiation markers filaggrin and loricrin in the *stratum corneum*. Seeding 61 years old donor's NHK over these DEs led to an obvious decrease in epidermal thickness (Figure 5b). Only little perlecan was found in the DEJ while laminin 332 was still expressed. The reduction in cell layers was accompanied by anomalies in the differentiation markers staining (Additional file 5: Figure S5), reflecting disorganization and abnormalities in the differentiation process. These data show that aged skin keratinocytes are unable to form a multi-layered well-differentiated epidermis in three-dimensional culture conditions. To address whether the defect in epidermal formation with aging is influenced by perlecan expression, we epidermalized SEs with the 61 years old donor's NHK in the presence of purified human perlecan. Perlecan was immunopurified from the conditioned medium of human coronary arterial endothelial cells as described previously [23] (Figure 5c and d). Epidermis made from aged skin NHK was thin and poorly organized, reaching a 50 μm depth at day 21 (Figure 5e and f). Exogenous addition of perlecan at 5 μg/ml (+ perlecan), restored the ability of aged NHK to form a multi-layered epidermis, which was already visible at day 14 and reached 100 μm thickness at day 21, where differentiation layers were readily distinguishable (Figure 5e and f). An additional application of perlecan (++ perlecan) further enhanced this phenomenon as evidenced by the 150-μm epidermal thickness obtained at day 21. Perlecaninduced improvement of the epidermal differentiation process was apparent after HPS staining (Figure 5h), and we analysed the differentiation markers expression at day 21 (Figure 5g). Interestingly, the K5 staining revealed that the basal keratinocyte layer was enlarged. The early differentiation marker K10 was expressed in all suprabasal layers, indicating that a normal differentiation process occurs in the perlecan-restored epidermis. Staining of the intermediate differentiation marker involucrin revealed a large multilayer zone containing well delineated and organized cells, in contrast to those found in the flat and packed epidermis of the control SE. Staining of filaggrin in the spinous layer and loricrin in the *stratum corneum* both revealed thicker sections containing healthy and actively producing cells compared to the thin and poorly organized layers obtained in the control SE. These results suggest that supplementing SE with perlecan allowed the aged skin keratinocytes to accomplish each differentiation phase, whereas they seemed incomplete and cell-deprived in the control SE. Staining of collagen VII and laminin 332 revealed a regular BM below the epithelium of both models, appearing thicker and less interrupted in the perlecan-treated SEs (Figure 5h). The addition of perlecan modified the β1 and α6 integrin patterns both in intensity and localization (Figure 5h); suggesting that perlecan favoured keratinocyte cohesion in the basal layer. The increase in K5-positive cells in perlecan-treated SEs suggested increased proliferation of basal cells. With Ki-67 staining, we found that the abundance of Ki-67-positive cells in the basal layer was 24% higher in the perlecan-treated SEs than in untreated SEs (Figure 5i). This result suggested that adding soluble perlecan enhanced the proliferation of basal cells. An examination of perlecan expression revealed stronger staining in the BM of treated SEs than in the BM of untreated SEs (Figure 5j). This result showed that some of the added perlecan reached the BM. However, a punctuated, circular staining pattern was observed throughout the epidermis, which suggested that some perlecan was trapped between keratinocytes. Analysis of K15 expression revealed that K15- expressing cells were present in the basal layer of perlecan-treated SE, but only a few were observed in untreated SE (Figure 5k). As previously documented, the presence of Ki-67-positive cells in the K15-positive basal layer indicated that this layer maintained a healthy, self-renewing, proliferative compartment [24].

## DISCUSSION

In this study, we examined the expression profile of perlecan during chronological skin aging and showed that its expression decreased over time. Perlecan sensitivity to proteolysis has suggested that controlled degradation processes may regulate its bioactivity, including growth factor storage and release [25,26]. Enzyme up-regulation during aging [26] or in senescent cells [27] may accelerate perlecan degradation. Our QPCR analysis revealed a reduced level of perlecan transcripts in aging keratinocytes, which suggested that disturbing the ability to neo-synthesize perlecan may create a disorder in the perlecan synthesis/degradation balance. This defect in perlecan synthesis was clearly observed in our SE models, where little perlecan was detected in the BM of models epidermalized with aged skin keratinocytes. The reduction in perlecan mRNA levels with aging may result from the location of its gene, namely *HSPG2,* on the telomeric portion of chromosome 1 at locus 1p36.1 [28]. Telomerase has been detected in basal keratinocytes and in the bulge component of the hair follicle [29]. Evidence suggests that telomeres shorten with age in human skin [30]. Our donor cohort included women only; thus, perlecan expression levels may have also been influenced by the sex hormone status of the donors, as suggested in an immunohistological study [31].

Perlecan is involved in the regulation and maintenance of epidermal BM integrity at various stages, including morphogenesis and repair. Perlecan was shown to exert its biological effects through its ability to bind growth factors and modulate their bioavailability and biological activities [12]. This property was brought to light in skin by Sher *et al*. [10], who showed that epidermal perlecan could control survival and terminal differentiation through a mechanism involving its binding to FGF7. In addition, HS moieties in HSPGs play a crucial role in growth factor storage and activity; this property was exemplified in a recent study, which showed that the overall degradation of HS at the DEJ caused abnormal epidermal differentiation [32]. By assembling *in vitro* skin models, we showed that aged keratinocytes, which were deficient in perlecan production, displayed an inability to form a multilayered epidermis. Addition of purified perlecan to the culture medium increased epidermal thickness and restored the morphology to a well-differentiated, multilayered epithelium. Basal and intermediate epidermal layers were particularly well developed, which confirmed the continuation of the stratification program. The presence of Ki-67 positive cells in the K15-positive basal layer demonstrated that this layer maintained the self-renewal capacity of basal keratinocytes, characteristic of keratinocyte stem cells [24]. A similar observation was documented in a recently published analysis of the corneal epithelium in perlecan-deficient mice [33]. The corneal epithelium of perlecan knockout mice was thin and poorly differentiated, displaying defects in cell proliferation and differentiation. Addition of exogenous perlecan to our skin model resulted in improvements of both cell proliferation and differentiation; indeed, keratin K5/K14-positive cells, known as proliferative cells, were particularly abundant. Interestingly, a recent study showed that exogenous, but not endogenous, perlecan induces keratinocyte proliferation *in vitro* [34]. Keratins K5, K14, and K15 are expressed in the basal layer of the epidermis, which contains epidermal stem cells and transit amplifying cells. K5 and K14 are prototypic markers of dividing basal keratinocytes, and they contribute to the maintenance of the epidermal cell shape [35,36]. As keratinocytes move upward and differentiate, basal keratin expression gradually decreases, and expression of the K1/K10 pair and involucrin is induced [Figure 3d, 37]. As illustrated in our perlecan immunofluorescence studies, part of the added exogenous perlecan reached the BM. To elucidate whether perlecan might directly impact the basal keratinocyte phenotype, we investigated its cell adhesion properties. Although involvement of perlecan in keratinocyte adhesion was recently suggested [34, 38], we found that perlecan induces keratinocyte adhesion through a Δ1 integrin mediated mechanism. Plating keratinocytes on perlecan reduced the expression of mRNAs that encoded early and intermediate differentiation markers (K1, K10, and involucrin). However, the expression of mRNAs that encoded the K5/K14 pair remained unchanged, mRNA expression of the basal keratin K15 was significantly increased. In contrast, an increase in K15 expression was not observed when cells were plated on laminin 332; demonstrating that the perlecan effect was specific. In addition, the effect was not observed when the perlecan HS moities were removed suggesting that intact perlecan was required for this mechanism. K15, which is specifically expressed in the basal cells of most stratified epithelia, was reported to be a marker of stem cells in the human epidermis [for review, see 39]. Moreover, in situ hybridization studies revealed that K15 transcription was down-regulated when postmitotic cells moved out of the basal layer [19]. Moreover, K15 protein and mRNA expression levels were suppressed under hyperproliferating conditions [40,19]; indeed, it has been suggested that the loss of K15 in human hair follicle stem cells was one of the earliest signs of the transition from a stem cell to a transit amplifying cell [41]. Therefore, our results suggested that perlecan may postpone the commitment of K15-expressing cells to the differentiation program. This result was further supported by the enhancement of Δ1-integrin subunit mRNA expression in perlecantreated cells. Furthermore, our findings were strongly reinforced by the fact that down-regulation of perlecan in keratinocytes with specific siRNAs resulted in depletion of the keratin 15/Δ1-integrin cell population. Previous results have shown that K15 was expressed in 50-70% of interfollicular basal keratinocytes in newborn skin, and this expression was only 20-30% in young children and adults, due to its restricted localization to the rete-ridges [21]. The undulating pattern of rete ridges becomes flattened in aged human skin, and the expression of some stem cell markers is reduced [18,42,43], which results in a reduced potential to self-renew *in vitro*. We showed here that aged skin displayed a reduced, punctuated K15 expression pattern, restricted to the basal pole of keratinocytes. The mechanism underlying perlecan maintenance of K15- positive epidermal keratinocytes may be linked to its pivotal role in BM assembly. Biochemical studies of the DEJ revealed its bridging function of the laminins 511/521 and −332 containing suprastructure with the collagen IV containing network [11]. Besides, perlecan was shown to support additional molecular interactions throughout the BM evidencing, together with nidogens, a role for creating molecular bridges compatible with cellular interactions [44]. This is of particular relevance as laminins 511/521 expressions correlated with the increased ability of keratinocytes to regenerate epithelium [45]. Therefore, we can speculate for a role of perlecan in recruiting and maintaining laminins 511/521, produced by keratinocytes and dermal pericytes [46], within the basal keratinocyte microenvironment. That perlecan governs proper development and maintenance of tissue architecture in tissue regeneration was recently exemplified in metazoan Nematostella vectensis [47]. Our results implied that the mechanism involved the perlecan HS moities, which also suggested the involvement of a growth factor. Although the molecular mechanisms that control K15 gene expression remain unknown, previous studies have revealed that K15 transcription appears to be suppressed by FGF7, TGF-Δ1, tumour necrosis factor alpha (TNF-α), and EGF in HaCaT cells [20]. Conversely, the thyroid hormone and interferon-gamma were shown to activate the K15 promoter [48]. Our study revealed a perlecan-dependent HB-EGF effect on K15 expression in keratinocytes, at both the mRNA and protein levels. We also observed a concomitant inhibition of keratinocyte proliferation, which corroborated a previous study that showed reduced cell growth in keratinocytes induced to overexpress HBEGF [49]. In that study, HB-EGF-overexpressing keratinocytes displayed down-regulated expression of the suprabasal epidermal keratins, K1 and K10 [49], a phenotype similar to the one we observed after culturing keratinocytes on perlecan. HB-EGF-mediated down regulation of K10 or suprabasal keratin expression was also observed in migrating epidermal margins during wound healing [50]. Taken together, these results suggested that perlecan drives HB-EGF regulation of the interfollicular self-renewing cells in the basal layer. Immunodetection of perlecan in cultured keratinocytes revealed its cell-associated localization; however, whether K15 regulation is the result of a direct integrin mediated interaction with perlecan remains to be demonstrated. Numerous *in vitro* and *in vivo* studies have highlighted the biochemical and biological roles of cell-associated perlecan in growth factor signalling [51]. Perlecan HS moieties were found to participate in the presentation of growth factors to their high-affinity receptors [52,53]. HB-EGF expression is restricted to the basal epithelium in normal skin [54]. Accordingly, it is tempting to suggest that, when combined with perlecan, HB-EGF may play a role in maintaining the K15-expressing sub-population of basal keratinocytes. Therefore, the decrease in perlecan expression observed during aging may impact the K15-expressing selfrenewing cells and alter epidermal homeostasis. When we supplemented the BM of our SEs with purified perlecan, the enhanced perlecan content correlated with the increase in K15 and Ki-67-positive cells in the basal layer. Our results that showed that HB-EGF induced proliferation in perlecan knock-down keratinocytes are puzzling; however, these results recalled findings from another study that described the autocrine-stimulating role of HB-EGF on keratinocyte proliferation [54]. Interestingly, transgenic mice that overexpressed the soluble form of HB-EGF under the control of the keratin 5 promoter exhibited severe epidermal hyperplasia [55]; that phenotype resembled the phenotype of the SEs we treated with perlecan supplementation. In our model, soluble HB-EGF produced by cells of the epidermal or dermal compartment, or contained in the culture medium may have been captured by perlecan HS, due to its heparinbinding domain. This could have increased the local HB-EGF concentration in the keratinocyte environment. These observations suggested that HB-EGF may trigger different signalling events, depending on how it is delivered: in a soluble form or associated with immobilized, cell-associated perlecan. Most interestingly, K15 expression in the basal layer of keratinocyte derived epithelial sheets was reported to depend on a factor produced by dermal fibroblasts [45]. In conclusion, our work provided evidence to support the notion that keratinocyte-associated perlecan might contribute to basal keratinocyte phenotype maintenance by down-regulating the expression of early and intermediate differentiation markers and up-regulating the expression of K15. This finding was particularly important, because K15-expressing keratinocytes are typically considered to be quiescent stem cells [56, 45]. Our work further revealed that HB-EGF, a growth factor produced by basal keratinocytes, up-regulated K15 expression in a perlecan-dependant manner. This result suggested that HB-EGF could play a role in regulating the K15-expressing stem cell sub-population. However, additional work must be performed to characterize whether other stem cell markers are regulated in this way. Nevertheless, our work suggested that cell-associated perlecan could be considered to play a role in the epidermal stem cell niche. Most interestingly, a recent study revealed that perlecan played a critical role in the regulation of intestinal stem cell activity by mediating the stem cell-ECM attachment [57]. Growth factors and cytokines in niches instruct stem cells to self-renew and maintain normal cycling status. That niches weaken with age, due to reduced expression of cell adhesion molecules and factors that control stem cell self-renewal, may profoundly affect the self-renewing properties of the epidermis basal layer.

## METHODS

### Collection of skin specimens

Full-thickness UV unexposed skin biopsies were obtained from female patients undergoing mammary or abdominal reduction following ethical and safety guidelines according to French regulation donors (Declaration no. DC-2008-162 delivered to the Cell and Tissue Bank of Hospices Civils de Lyon). Thirty-eight subjects were enrolled in this study: 18 elderly donors (ages 50–73), 12 intermediate donors (ages 35–49) and 8 young donors (age 22-34). After surgery, tissues were embedded in Tissue-Tek OCT compound (Euromedex, Mundolsheim, France) and kept at −80°C, processed for paraffin embedding or handled for cell extraction.

### Monolayer cell cultures

NHK cultures were established from skin specimens according to previously published procedures [58] and grown in supplemented keratinocyte growth medium containing 0.15 mM CaCl_2_ (KBM-2 BulletKit, Lonza Biosciences, Basel, Switzerland). Fibroblasts were grown in Fibroblast Growth Medium (Promocell, Heildeberg, Germany). Keratinocytes used in SEs were isolated and cultured as described by Rheinwald and Green [59] on a feeder layer of irradiated human fibroblasts as previously described [22].

### Preparation of skin equivalents

Fibroblasts (passage P7, 25 × 10^4^ cells/cm^2^) were seeded onto a dermal substrate and grown for 21 days as described previously [22]. Keratinocytes were seeded on the DE at a density of 25 × 10^4^ cells/cm^2^ in a 3:1 DMEM:Ham's F12 (Gibco, Life Technologies SAS, Saint Aubin, France) supplemented medium [22]. After 7 days, the SE was elevated to the air–liquid interface and cultured in a differentiation medium. SEs were cultured for up to 42 days, and were harvested at 21, 35, or 42 days after fibroblast seeding. SEs were either fixed in neutral buffered formalin 4% (Diapath, Martinengo, Italy) for 24 h and embedded in paraffin or in OCT compound and frozen at −80°C. When indicated, 5 μg/ml of human purified perlecan was added to the SEs culture at different time points. The first application was on top of DEs before keratinocytes seeding. Following incubation at 37°C for 16 h, keratinocytes were seeded in medium containing 5 μg/ml perlecan. After 2 days, the medium containing 5 μg/ml perlecan was either renewed once (namely + perlecan) or twice (namely ++ perlecan) and the normal culture conditions were maintained thereafter. All SEs were produced in triplicate and each experiment was repeated twice.

### Western blot analysis and perlecan purification

Keratinocyte ECM was obtained as described previously (Décline and Rousselle, 2001). Proteins were resolved on SDS-PAGE gradient gels (3-5%) and perlecan was immunoblotted as previously described [38]. Perlecan was purified by ion exchange and mAb affinity chromatography from human coronary arterial endothelial cell conditioned medium [23]. To ensure removal of growth factors bound to perlecan, the affinity chromatography column was extensively washed with 0.02 M Tris-HCl, 1M NaCl, pH 7.4, before perlecan elution. Prior to SDS-PAGE analysis, aliquots were TCA precipitated and analysed either by western blotting or by staining of the gel with Coomassie Brilliant Blue. HS digestion was performed as previously described using 8 mU/ml heparitinase I for 2 h at 25°C [60].

### Histological and immunohistological procedures on tissues

Paraffin sections were processed either for Haematoxylin – Phloxin – Saffron (HPS) staining or for immunohistochemical experiments. Sections were dewaxed, antigen retrieval was performed according to the manufacturer's instructions and immunolabelling experiments were performed, using antibodies against: perlecan (3G166 against domain III, US Biologicals, Swampscott, USA); keratin 10 (DE-K10, Dako), Δcatenin (E-5, Santa Cruz Biotechnology, distributed by Clinisciences, Montrouge, France); laminin 332 [61] and collagen VII (Santa Cruz Biotechnology). Other mAbs against perlecan (clone 6F165 against domain I, clone A7L6 against domain IV, and clone 6F166 against domain V from US Biologicals) were applied on 10 μm thick frozen sections after fixation in 4% paraformaldehyde in PBS and incubation in 20 mM sodium acetate, pH 7.0, 5 mM CaCl_2_ containing 2 mIU/ml heparitinase I and 10 mIU/ml chondroitinase ABC for 30 min at 25°C (Seikagaku America, distributed by Coger, Paris, France). After incubation with 5% normal goat serum in PBS, peroxidaseconjugated antibodies (En Vision dual-link, Dako, Trappes, France) were used. Counterstaining was performed using Harris haematoxylin (25%, Sigma Aldrich, Saint-Quentin-Fallavier, France).

Frozen sections were fixed in pre-cooled acetone for 10 min and rehydrated with 10% FCS in PBS. After washing, sections were incubated overnight with Abs against keratin 5 (D5/16B4, Dako), filaggrin (15C10, Vector Labs, distributed by Clinisciences), involucrin (BT-600, BTI, Clinisciences), loricrin (15C10, Vector Labs), Δ1 (P4C10, Millipore, Molsheim, France) and α6 (GoH3, Millipore) integrin subunits mAbs or Ki-67 (clone MIB-1, Dako), and washed. For K15 staining, frozen section were fixed in ethanol 70% and blocked. Anti-K15 pAb [62] (EPR1614Y, Gene Tex distributed by tebu-bio SAS, Le Perray en Yvelines, France) was applied 1 h and rinsed. Cy3 (Jackson, Beckman Coulter, Paris) or alexa-488 (Molecular probes, Invitrogen) conjugated antibodies were applied for 1 h. Nuclei were stained with DAPI. Fluorescence was visualized using LSM700 laser scanning confocal microscope (Zeiss, Jena, Germany).

### Image analysis and processing

Perlecan quantification for the skin cohort study and epidermal thickness measurements were performed using ImageJ software. Brightfield images of DAB-labelled perlecan and haematoxylin counterstaining were converted to normalized blue images that allow automated identification of positively stained tissue according to [63]. Epidermal thickness of HPS-stained sections was obtained by means of an Euclidean distance map. Pixels corresponding to the epidermis were isolated from other pixels. Images were converted to 8-bit binary images. Images corresponding to the area of interest were converted to 16-bit distance maps. To each epidermis pixel (nonzero) in the distance map binary image a value equal to its distance from the nearest background pixel (zero) was assigned. The basal epidermal line was selected and then applied on the distance map. The mean intensity of the basal line corresponds to the mean distance between the basal line and the *stratum corneum*. Twelve different fields per experimental condition were quantified. Data are expressed in μm.

### Immunofluorescence studies on cells

NHEK from young and aged donors were grown on chambered coverglasses in supplemented KBM-2. After washing, cells were fixed with 4% paraformaldehyde in PBS, permeabilized with 0.1% Triton X-100 for 2 minutes and incubated with 1% BSA / PBS. Anti-perlecan pAb (Santa Cruz) or anti-K15 [57], (EPR1614Y, Gene Tex distributed by tebu-bio SAS, Le Perray en Yvelines, France) were applied. Alexa Fluor 488- and/or Alexa Fluor 568-antibodies were applied, either alone or with fluorescein isothiocyanate-phalloidin (FITC; Sigma). Nuclei were stained with DAPI. Cells were observed with LSM700 laser scanning confocal microscope.

To quantify perlecan in cell cultures, NHEK were seeded in 96-well plates and grown until subconfluence. The same culture procedure as described above was applied except that wells were incubated in digestion buffer containing 2 mIU/ml heparitinase I and 10 mIU/ml chondroitinase ABC prior to the saturation step with BSA 1% and subsequent incubation with antiperlecan antibodies. Next, culture plates were washed with PBS and incubated with FITC-conjugated secondary antibodies for 1 h. After PBS washes, 20 mM NH4OH, Triton 0.5% was added to each well and the fluorescence intensity determined at 485-535 nm using a VICTOR X4 Multilabel plate reader (Perkin Elmer SAS, Courtaboeuf, France). A blank value corresponding to BSA-treated wells was subtracted and each assay point was measured 6 times. Proliferation assays were performed using Cell Proliferation Kit XTT (Roche Diagnostics).

### Quantitative real-time PCR analysis

Total RNA was isolated from monolayers using RNA isolation NucleoSpin RNA XS (Macherey-Nagel EURL, Hoerdt, France) according to the manufacturer's instructions. 500 nanograms of total RNA extracted from young and aged NHK cells were subjected to the first strand cDNA synthesis using a mix of oligo dT and random 6-mers using the PrimeScript RT reagent Kit (Perfect Real Time, TAKARA Biotechnology, distributed by OZYME, Montigny Le Bretonneux, France). QPCR was performed using the FastStart Universal SYBR Green Master kit (Roche Diagnostics, Mannheim, Germany). The primers used for the detection of gene expression are listed below. Reactions were performed using a Qiagen real-time PCR cycler Rotorgene. The presence of a single dissociation peak was verified by melt curve analysis. PCR end products were separated on a 2% agarose gel to confirm the amplification of single bands of the expected size. The specificity of target amplification was confirmed by sequencing. Relative quantification was determined using the comparative C(t) method [64] with normalization to the housekeeping gene GAPDH. Each assay point was performed in triplicate.

### Cell adhesion assays

Perlecan was immobilized on multiwell plates (Greiner, Dutscher, Brumath, France) as indicated concentrations by overnight adsorption at 4°C. Plates were washed with PBS, and saturated with 1% BSA. Next, keratinocytes were detached with trypsin, suspended in supplement-free KBM-2 basal medium (0,15 mM CaCl_2_) and seeded at 3 × 10^4^ cells/well. For cell adhesion inhibition experiments, the anti-Δ1 integrin antibodies (P4C10, Millipore, Molsheim, France) were added to the cells at the time of seeding. After 1 h, non-adherent cells were removed with a PBS wash. The extent of adhesion was determined by fixing adherent cells, then staining with 0.1% crystal violet and measuring absorbance at 570 nm as previously described [65]. A blank value was subtracted that corresponded to BSA-coated wells. Each assay point was derived from six measurements ± SD (6 wells per assay point).

### Growth factor treatments

10^5^ keratinocytes were cultured in a 24 well plate for 16-20 hours in KBM-2. They were rendered quiescent by serum starvation for 16 to 20 h and subsequently treated with fresh supplement-free KBM-2 medium containing 10 ng/ml FGF7, 50 ng/ml IGF-1, 80 ng/ml IGF-2, 100 ng/ml HBEGF or 1ng/ml TGF β1 respectively (Pepro Tech France, Neuilly sur Seine, France), for 5 hours. Cells were harvested and counted or lysed with PBS 1% Triton prior to K15 western blotting experiments or for RNA isolation.

### SiRNA experiments

For human perlecan knock-down experiments, a pool of 3 perlecan specific siRNAs was used (Perlecan siRNA, Santa-Cruz Biotechnology). Cell transfection was performed using Lipofectamin 3000 reagent (Invitrogen, Cergy Pontoise, France) with 100 nM duplex siRNA according to the manufacturer's recommendations. In all experiments, cells were analysed 48h after transfection and viability was assessed using trypan blue. A firefly luciferase GL2 sequence (CGUACGCGGAAUACUUCGA) was used as a negative control (100 nM), as described [66], and resulted in no reduction of perlecan expression.

### Statistical analysis

Data were presented as mean ± SD and the Student's t-test (unpaired, two-tailed) was used for two-group comparisons. The exact sample size (n) for replicate measurements of a specific keratinocyte strain or of groups of keratinocytes from distinct donors are specified in each graph legend. Individual data values are shown when n < 6 according to [67]. Differences were considered statistically significant at a value of P <0.05.

## SUPPLEMENTAL FIGURES



## Abbreviations

DEJ: dermal-epidermal junction
ECM: dermalextracellular matrix
BM: basement membrane
HSPG: heparan sulfate proteoglycan
NHK: normal human keratinocytes
SE: skin equivalent
DE: dermal equivalent
