# Supplementary material for: Perlecan expression influences the keratin 15‐positive cell population fate in the epidermis of aging skin
Source: Aging (Albany NY). 2016 Mar 17;8(4):751–68. doi: 10.18632/aging.100928 (PMC4925826; doi:10.18632/aging.100928)
Supplement: Supplementary file 1 [file aging-08-751-s001.pdf]

SUPPLEMENTARY FIGURES

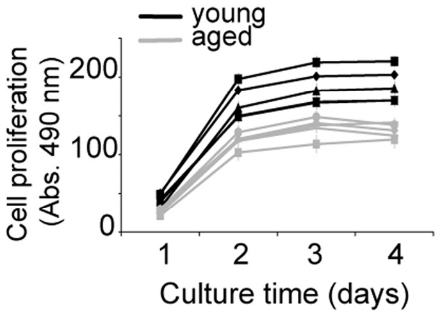

Figure S1. Proliferation of keratinocytes from young and aged donors. NHKs (5 × 103) derived from donors aged 22, 24, 26, 27, and 32 years or 52, 55, 56, 61, and 64 years were seeded in 96-well plates, and proliferation was monitored every day over 4 days with the XTT assay. Each assay point was performed at least 6 times.

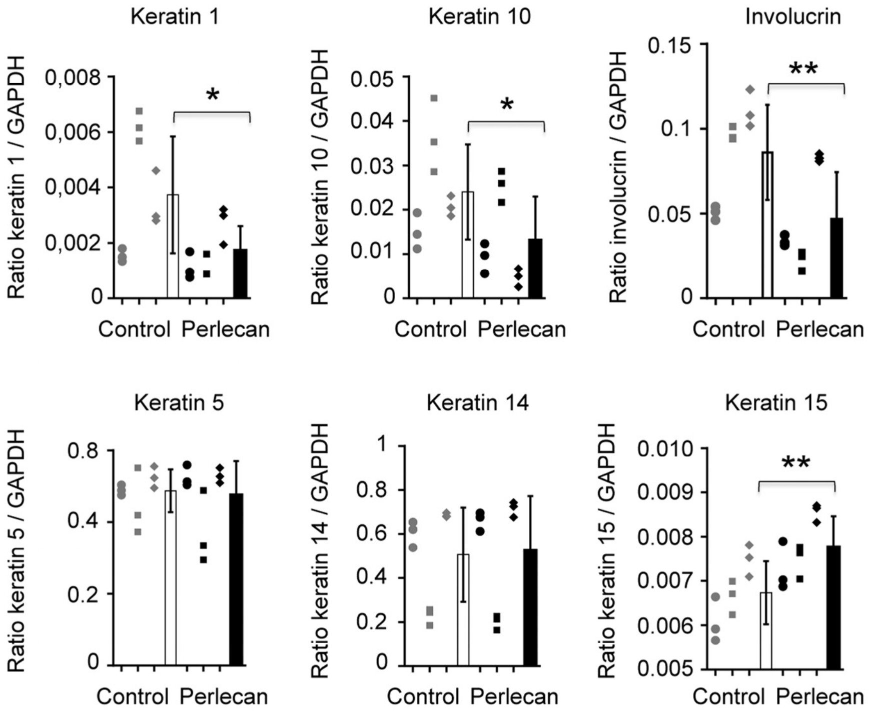

Figure S2. Perlecan effect on keratins and involucrin gene expression in aged keratinocytes. Real-time PCR analysis of keratins (K1, K5, K10, K14, and K15) and involucrin gene expression normalized to GAPDH in keratinocytes from 3 distinct aged donors (age 64, 61, 60), plated on culture dishes either covered beforehand with perlecan or left untreated. Histograms show the mean ± SD of 9 independent experiments conducted with keratinocytes from distinct donors. *p<0.025,**p<0.005,***p<0.0002 vs. control, Student's t-test.

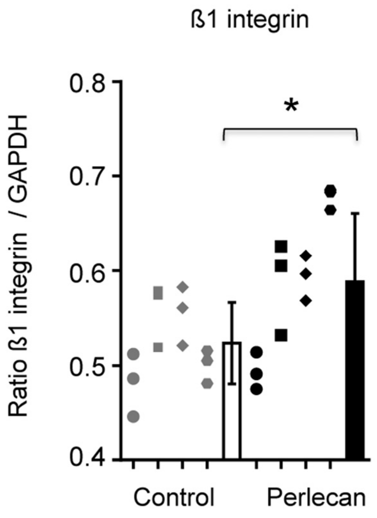

Figure S3. Perlecan effect on β1-integrin gene expression in aged keratinocytes. Real time PCR analysis of β1-integrin gene expression normalized to GAPDH in keratinocytes from 4 distinct aged donors (age 64, 61, 60, 49), plated on culture dishes either covered beforehand with perlecan or left untreated. Histograms show the mean ± SD of 12 independent experiments conducted with keratinocytes from distinct donors. *p<0.025 vs. control, Student's t-test.

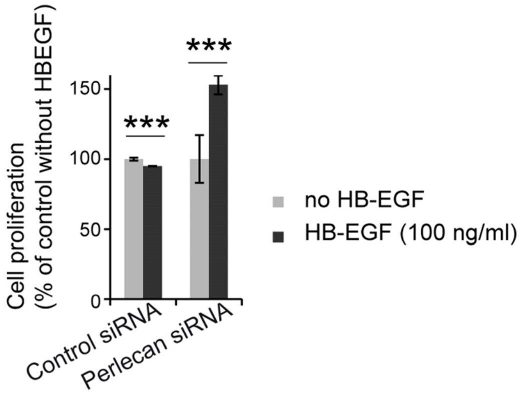

Figure S4. Perlecan-dependant effect of HB-EGF on cultured keratinocytes. Impact of HB-EGF (100 ng/ml) on the proliferation of a 52-year old keratinocyte strain treated with control siRNA or perlecan-siRNA beforehand. Each assay point was performed 12 times. Data are presented as the mean ± SD with keratinocytes from the same donor. ***p<0.0002 vs. control, Student's t-test.

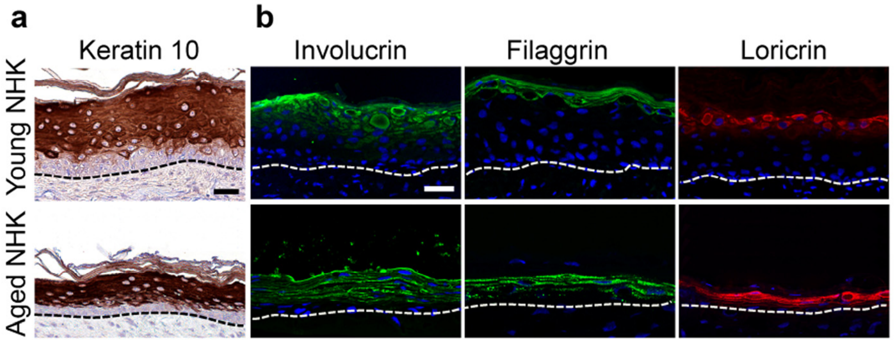

Figure S5. Aged keratinocytes display differentiation disorders in skin equivalent models. Human dermal fibroblasts isolated from skin donors aged 20 years were seeded on a dermal substrate. After 21 days in culture, NHKs from skin donors aged 20 or 61 years were seeded on each DE and cultivated for up to 42 days. (**a**) Cross-sections of paraffin-embedded SEs that were immunolabelled for keratin 10. (**b**) Frozen sections were probed for involucrin, filaggrin, and loricrin. Nuclei were stained with DAPI. Scale bars = 50 µm.
